# MiR-216b inhibits cell proliferation by targeting FOXM1 in cervical cancer cells and is associated with better prognosis

**DOI:** 10.1186/s12885-017-3650-5

**Published:** 2017-10-04

**Authors:** Shanyang He, Bing Liao, Yalan Deng, Chang Su, Jiuling Tuo, Jun Liu, Shuzhong Yao, Lin Xu

**Affiliations:** 1 Department of Obstetrics and Gynecology, the First Affiliated Hospital, Sun Yat-sen University, Guangzhou, 510700 China; 2 Department of Pathology, Zhongshan School of Medicine, Sun Yat-sen University, Guangzhou, China; 3 Department of Hematology, the First Affiliated Hospital, Sun Yat-sen University, Guangzhou, China; 4 Department of Microbiology, Zhongshan School of Medicine, Sun Yat-sen University, Guangzhou, 510080 China

**Keywords:** Cervical cancer, FOXM1, microRNA, Proliferation, Prognosis

## Abstract

**Background:**

Our previous study showed FOXM1 expression was significantly up-regulated in cervical cancer, and was associated with poor prognosis. To clarify miRNAs-FOXM1 modulation pathways, in this study, we investigated the relationships between miR-216b and FOXM1 and the role of miR-216b in cell proliferation and prognosis of cervical cancer patients.

**Methods:**

Western blotting and qPCR were used to determine expression of FOXM1, cell cycle related factors and miR-216b level. MiR-216b overexpression and inhibited cell models were constructed, and siRNA was used for FOXM1 silencing. Cell proliferation was analyzed by MTT and colony formation assay. Dual luciferase reporter assay system was used to clarify the relationships between miR-216b and FOXM1. Kaplan-Meier survival analysis was used to evaluate prognosis.

**Results:**

MiR-216b was down-regulated in cervical cancer cells and tissues, and its ectopic expression could decrease cell proliferation. Western blotting analysis showed miR-216b can inhibit cell proliferation by regulating FOXM1-related cell cycle factors, suppressing cyclinD1, c-myc, LEF1 and p-Rb and enhancing p21 expression. Repressing of miR-216b stimulated cervical cancer cell proliferation, whereas silencing FOXM1 expression could reverse this effect. Western blotting and luciferase assay results proved FOXM1 is a direct target of miR-216b. Survival analysis showed higher level of miR-216b was associated with better prognosis in cervical cancer patients.

**Conclusions:**

FOXM1 expression could be suppressed by miR-216b via direct binding to FOXM1 3′-UTR and miR-216b could inhibit cell proliferation by regulating FOXM1 related Wnt/β-catenin signal pathway. MiR-216b level is related to prognosis in cervical cancer patients and may serve as a potential prognostic marker.

**Electronic supplementary material:**

The online version of this article (10.1186/s12885-017-3650-5) contains supplementary material, which is available to authorized users.

## Background

Cervical cancer is the third most frequent cancer and the fourth leading cause of cancer death in females worldwide, and more than 85% of these cases and deaths are in developing countries [1], despite the advances in screening and early diagnostic methods in recent years [2]. By now, the molecular mechanisms of tumor aggressiveness of cervical cancer still remain to be elucidated and more tumor-specific markers for molecular therapy need to be confirmed. In previous study, we focused on a tumor-related transcription factor FOXM1, and explored its role in cervical cancer metastasis. We found that enforced expression of FOXM1 could increase growth, migration and invasion ability of cervical cancer cells [3], and clinical retrospective study showed that overexpression of FOXM1 could serve as an independent prognostic factor for poor survival in patients with early-stage cervical cancer [3]. Therefore, FOXM1 could act as a prognostic marker of cervical cancer, and a promising tumor-specific marker which has potential application value in molecular intervention therapy. However, its upstream regulation pathway and modulation molecule needs to be elucidated.

MicroRNAs are endogenous, single-stranded, small non-coding RNAs that post- transcriptionally modulate gene expression involved in essential cellular processes. They guide the binding of RNA-induced silencing complexes to partially complementary regions located usually within the 3′ untranslated regions of target messenger RNAs (mRNAs), thus resulting in target mRNA degradation and/or translational inhibition [4–6]. Aberrant expression of miRNAs has been found in different types of cancers, and some of them function as tumor suppressor genes (e.g. miR-29c and miR-125b, etc.), whereas some act as oncogenes (e.g. miR-151 and miR-454, etc.) [4–9]. Recently, some microRNAs has been proved to modulate FOXM1 expression in many cancers [10], including hepatocellular carcinoma [11], breast cancer [12], gastric cancer [13], colorectal cancer [14], bladder cancer [15], squamous cell carcinoma [16], lung cancer [17, 18], leukemia [19], etc. But little is known about the miRNAs-FOXM1 signaling pathways that modulate the pathogenesis and progression in cervical cancer patients.

In this study, we detected the miR-216b level in different cervical cancer cell lines, and found that miR-216b level negatively correlated with the FOXM1 expression. Functional assay demonstrated that miR-216b could inhibit the proliferation of cervical cancer cells by down-regulating p-Rb, c-myc and cyclinD1, which were downstream targets or important regulators of FOXM1. Further studies found that miR-216b could bind the 3′-UTR of FOXM1 and inhibit FOXM1 expression. Therefore, we proved that FOXM1 was a direct and functional target of miR-216b, and like FOXM1, miR-216b may act as a prognostic marker of cervical cancer patients.

## Methods

### Patients

MiR-216b activity and target study enrolled 8 patients, who were diagnosed with early-stage cervical squamous cell carcinoma (SCC) and received radical hysterectomy and lymphadenectomy in the Department of Obstetrics and Gynecology, the First Affiliated Hospital, Sun Yat-sen University from January 2009 to December 2012. The enrollment criteria were SCC patients with no preoperative radiotherapy or chemotherapy and with clinical follow-up data. Clinical stage was determined according to the International Federation of Obstetrics and Gynecology, 2009 (FIGO). Totally 8 fresh cervical SCC samples and their corresponding tumor adjacent tissue samples were collected for determination of relative miR-216b expression level using quantitative polymerase chain reaction (qPCR). Samples of normal cervix from patients undergoing simple hysterectomy because of uterine leiomyomata were obtained as a control in FOXM1 western blotting and miR-216b qPCR analysis of 5 cervical cancer cell lines.

The survival and prognosis study of miR-216b enrolled 150 cervical cancer samples randomly collected from 2009 to 2012. The enrollment criteria were all the cervical cancer patients with pathological biopsy confirmation and clinical follow-up data, irrespective of the stage and patient age. Patients from Ia2 to IIa1 received radical hysterectomy and concomitant chemo-radiotherapy according to their risk factors. Patients with IIa2 and higher stage receive concomitant chemo- radiotherapy, or only follow-up. Of the collected cases, 121 were SCC and 29 were other types. The 150 samples were detected for their miR-216b expression using qRT-PCR. The results showed that among them, 75 were relatively miR-216b high level and 75 were miR-216b low. The mean age of these patients was 55.0 ± 10.3 (ranging from 29 to 75), and no age differences existed between miR-216b-high and miR-216b-low patients (*P* > 0.05). The last follow-up was carried out in December 2015, with the mean observation period of 41 months (1–60 months), and there were 95 cancer-related deaths. Prior written consent of each patient for the use of clinical materials for research purposes was signed, and approval from the Institutional Ethical Board (IRB) in the First Affiliated Hospital of Sun Yat-sen University was obtained. The clinical information of patients in survival analysis was summarized in Table 1.

**Table 1:** Clinicopathological characteristics and expression of miR-216 in studied cervical cancer patients

Factor	No.	(%)
Age (years)		
≤ 55	76	50.6
> 55	74	49.7
FIGO stage		
I/II	51	34
III/IV	99	66
Histology		
Squamous	121	80.7
Others	29	19.3
Survival status		
Alive	55	36.7
Dead	95	63.3
Expression of miR-216		
Low expression	75	50
High expression	75	50

### Cell lines and cell transfection

The cervical cancer cell lines HCC94 (Cat no. YB-ATCC-5495, FOXM1-low [3]), HeLa (Cat no. CCL-2), SiHa (Cat no. HTB-35, FOXM1-high [3]), Ca Ski (Cat no. CRL-1550) and C33A (Cat no. HTB-31) cell lines were obtained from the Department of Anatomy, the Zhongshan School of Medicine, Sun Yat-sen University, and cultured in RPMI 1640 medium (Gibco BRL, Rockville, MD). Media were supplemented with 10% fetal bovine serum (FBS, Gibco BRL, Rockville, MD) and 1% antibiotics mixture (100 U/ml penicillin and 100 μg/ml streptomycin) in a 5% CO2 humidified atmosphere at 37 °C [3, 20]. Medium was changed every 2 days. These five cell lines were all from American Type Culture Collection (ATCC, MD, USA).

MiR-216b mimics and mimics negative control (NC), miR-216b inhibitors (miR-216b-in) and negative control inhibitors (NC-in), mutant miR-216b and FOXM1-siRNAs were all synthesized by RiboBio. (RiboBio Co. Guangzhou, China). The concentration of miR-216b mimics and inhibitors was 20 nM, and in transfection, 2 μl/well of mimics/inhibitors or control mimics/inhibitors were added. Cells were inoculated into 6 well culture plate (Corning, NY, USA) at the concentration of 5 × 10^5^/ml the day before transfection, and cells were cultured in 2 ml/well of complete medium until 90% confluence. Transfection was performed by Lipofectamine 2000 (Invitrogen, Carlsbad, CA, USA) according to the manufacturer’s instructions. 48 h after transfection, total RNAs were prepared and used for qRT-PCR and the proteins were extracted for Western blotting immediately, or stored at −80 °C for future use.

### RNA extraction and qRT-PCR

Quantitative RT-PCR was used for the analysis of miR-216b expression level, and cyclinD1, myc and LEF1 (lymphoid enhancer-binding factor 1) mRNA level as described elsewhere [18–24]. Briefly, total RNA was extracted using TRIZOL Reagent (Invitrogen, CA, USA) from cultured cells following the manufacturer’s instructions. qRT-PCR was performed using iScript™ cDNA Synthesis Kit (Bio-Rad, Hercules, CA, USA) and SsoFast EvaGreen Supermix (Bio-Rad, Hercules, CA, USA) according to the manufacturer’s instructions. The miR-216b primers were synthesized by RiboBio Co., Guangzhou. The qRT-PCR procedure used to detect the miR-216b level was: cycle 1, 95 °C for 2 min; cycle 2 through 40, 95°Cfor 30 s, 60 °C for 35 s, and fluorescence signal was detected at the end of each cycle. Melting curve analysis was drawn to confirm the specificity. U6 snRNA level was used as an internal control for normalization. The primers used in *cyclinD1*, *myc* and *LEF1* mRNA detection were shown as follows. CyclinD1 forward: 5′-AACTACCTGGACCGCTTCCT-3′, reverse: 5′-CCACTTGAGCTTGTTCAC CA-3′. MYC forward: 5′-TCAAGAGGCGAACACACAAC-3′, reverse: 5′-GGCCTTTTCATTGTTTTCCA-3′. LEF1 forward: 5′-CACTGTAAGTGATGA GGGGG-3′, reverse: 5′-TGGATCTCTTTCTCCACCCA-3′. β-actin forward: 5′-TGGCACCCAGCACAATGAA-3′, reverse: 5′-CTAAGTCATAGTCCGCCTA GAAGCA-3′. Detection of each sample was repeated 3 times and the results were analyzed by Bio-Rad CFX96 Manager software.

### Construction of *FOXM1* 3′-UTR-PsiCHECK2 vector

The 3′ untranslating region (3′-UTR) of *FOXM1* containing putative miR-216b target binding sites was amplified by PCR from FOXM1 high-expression HeLa cells. The sequence of the *FOXM1* 3′-UTR forward primer was: 5′- CCGCTCGAGGGACTGTTCTGCTCCTCATAG-3′; and the reverse primer was: 5′- ATAAGAATGCGGCCGCTGGCAGTCTCTGGATAATGATC-3′. The primers contained *Xho I* and *Not I* restriction sites, respectively. The amplified 3′-UTR region was then subcloned into the *Xho I*/*Not I* sites of the PsiCHECK2 vector (Promega, Madison, WI, USA) behind the start codon and identified by sequencing, as described elsewhere [18, 23, 25]. The PCR procedure was: 94 °C 4 min, 1 cycle, 94 °C 30s, 62 °C 30s, 72 °C 30s, 35 cycles, 72 °C, 7 min.

### Western blotting analysis

Western blotting analysis was performed with standard techniques, as described previously [3]. Cell proteins were extracted by a modified RIPA buffer containing 0.5% sodium dodecyl sulfate (SDS) in the presence of a proteinase inhibitor cocktail (Roche, IN, USA). Polyacrylamide gel electrophoresis (PAGE) was performed to separate cell lysate proteins and then fractionated proteins were transferred onto a PVDF membrane (Amersham Biosciences, NJ, USA). Immonodetection was performed using antibodies including rabbit anti-FOXM1 polyclonal antibody, anti-cyclinD1, anti-p21, anti-LEF1, anti-c-myc, anti-Rb, anti- phosphorylated –Rb, and β-actin antibodies (Cell Signaling Technology, Danvers, MA, USA) at the dilution ratio of 1:1000. The membrane was then incubated with HRP labeled goat anti-rabbit secondary antibody (BosterBio, CA, USA) at the dilution ratio of 1:6000. Anti-β-actin (Cell Signaling Technology, Danvers, MA, USA) served as an internal control. Signals were detected by exposure to films with SuperSignal West Pico Chemoluminescent substrate (Thermo Fisher Scientific, MA, USA).

### Luciferase assay

For luciferase reporter assays, 5 × 10^5^ HeLa cells were transfected using Lipofectamine 2000 (Invitrogen, Carlsbad, CA, USA) in 24-wells culture plates, with 5 pmol of miR-216b (or mimics negative control, or miR-216b-mut), and 100 ng of firefly luciferase reporter vector in the transfection mixture. MiR-216b mimics negative control served as a negative control (NC) and microRNA inhibitor control served as NC-in control. Cells were harvested 48 h after transfection, and then the luciferase activity was measured using a dual luciferase reporter assay system (Promega, WI, USA) according to the manufacturer’s instructions. Three independent experiments were performed and the data were presented as the mean ± SD.

### MTT assay

Cell proliferation assay was performed using 3- (4, 5-dymethyl-2-thiazolyl) -2, 5- diphenyl-2H-tetrazolium bromide (MTT) assay, as described elsewhere [18, 23, 25]. Briefly, different groups of 2 × 10^3^ cultured HeLa cells were seeded into U-bottom 96-well plates per well (Corning, NY, USA) and cultured with miR-216b mimics and negative control (NC), miR-216b inhibitors (miR-216b-in) and negative control inhibitors (NC-in), mutant miR-216b and FOXM1-siRNAs respectively in 200 μl per well culture medium. Totally 4 duplicate plates were inoculated. Cultures were maintained for 7 days at 37 °C, 5%CO_2_ in a humidified atmosphere. Cell proliferation was detected on day 0–5 by MTT method and each group was analyzed in triplicate wells. MTT solution of 5 mg/ml (Sigma, MO, USA) was added at 20 μl per well during the final 4 h of culture. The medium was then replaced with 150 μl dimethyl-sulfoxide (DMSO) and vortexed for 10 min. The optimal density (OD) was read at a wavelength of 490 nm on a Tecan Sunrise microplate reader. Relative MTT absorbance was counted by: average OD_exp_ on day X/average OD_NC_ on day 1.

### Colony formation assay

Colony formation assay was performed as described elsewhere [18, 23, 25]. Briefly, different groups of 1 × 10^3^ HeLa cells were seeded into 6-well plates (Corning, NY, USA) per well and cultured with miR-216b mimics and negative control (NC), miR-216b inhibitors (miR-216b-in) and negative control inhibitors (NC-in) in 2 ml in RPMI 1640 medium supplemented with 10% fetal bovine serum (FBS). Cells were cultured for 7–10 days and colonies were observed everyday. The medium was removed and washed by PBS for 3 times. Cells were fixed by methanol for 10 min and stained with 0.1% crystal violet for 10 min. The numbers of colonies with more than 50 cells were counted manually.

### Statistical analysis

All statistical analyses were performed using SPSS 16.0 software package (SPSS Inc. IL, USA). The measurement data are expressed as mean ± standard error (Mean ± SD). The relationships between FOXM1 expression and miR-216b expression level were determined by correlation analysis and expressed as correlation coefficient (r). Differences of measurement data were assessed by Student’s *t* test. The clinicopathological differences between miR-216b-high and miR-216b-low patients were assessed using Pearson’s χ^2^ test. Survival curves were estimated using the Kaplan-Meier method. A two-sided value of *P* < 0.05 was considered statistically significant.

## Results

### MiR-216b expression was down-regulated in both cervical cancer cell lines and clinical samples

**Fig. 1:**
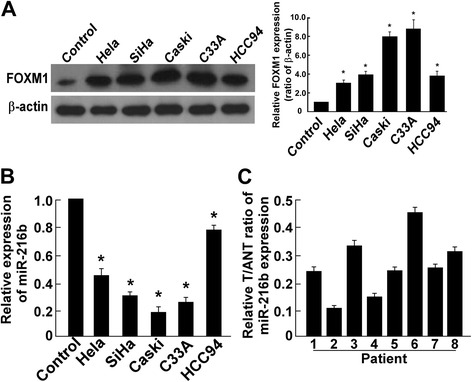
Expression of miR-216b showed an opposite trend against FOXM1 level in cervical cancer cells and was down-regulated in cervical cancer tissues. **a** Western blotting analysis showed FOXM1 expression levels were significantly elevated in cervical cancer cell lines HeLa, SiHa, Ca Ski, C33A and HCC94. Control was normal cervical tissue from patients underwent hysterectomy because of uterine leiomyomata. * *P* < 0.05. **b** Real-time PCR analysis of miR-216b expression in cervical cancer cell lines (HeLa, SiHa, Ca Ski, C33A and HCC94) showed an opposite trend compared to FOXM1. The average miR-216b expression was normalized using U6 expression. Control was tissue from normal cervix. * *P* < 0.05. **c** Relative T/ANT ratio showed miR-216b level was decreased in cervical cancer tissues than adjacent non-cancer tissues. The expression of miR-216b was examined in 8 paired cervical cancer tissues (T) and their adjacent non-cancer tissues (ANT). Relative T/ANT ratio was shown

FOXM1 and miR-216b level were screened in different cervical cancer cell lines, including HeLa, SiHa, Ca Ski, C33A and HCC94. FOXM1 expression was higher in Ca Ski, C33A and SiHa cells, and lower in HeLa and HCC94 cells (Fig. 1a). Quantitative RT-PCR showed that in Ca Ski, C33A, and SiHa cells, the miR-216b relative ratio was lower and in HeLa and HCC94 cells, miR-216b level was relatively higher (Fig. 1b). HeLa cells were selected for the following miR-216b study because this cell line showed better reaction to miR-216b mimics and inhibitors in preliminary tests and had moderate miR-216b and FOXM1 expression. In all the 5 cervical cancer cell lines, miR-216b level was significantly lower compared to the negative control (*P* < 0.05), indicating that miR-216b may be negatively related to cervical cancer tumorigenesis (Fig. 1b). Therefore, we further analyzed the relative T/ANT (cancer tissues/adjacent non-cancer tissues) ratio of miR-216b expression in 8 cervical cancer patients, and found that in all the cases, the T/ANT ratio was lower than 0.5, proving that in cervical cancer tissues, the miR-216b expression was dramatically down-regulated (Fig. 1c). These results showed that miR-216b level had an opposite trend of variation against FOXM1 expression, and suggested that miR-216b may be a negative regulator of cervical cancer.

### MiR-216b inhibits cell proliferation of cervical cancer cells

**Fig. 2:**
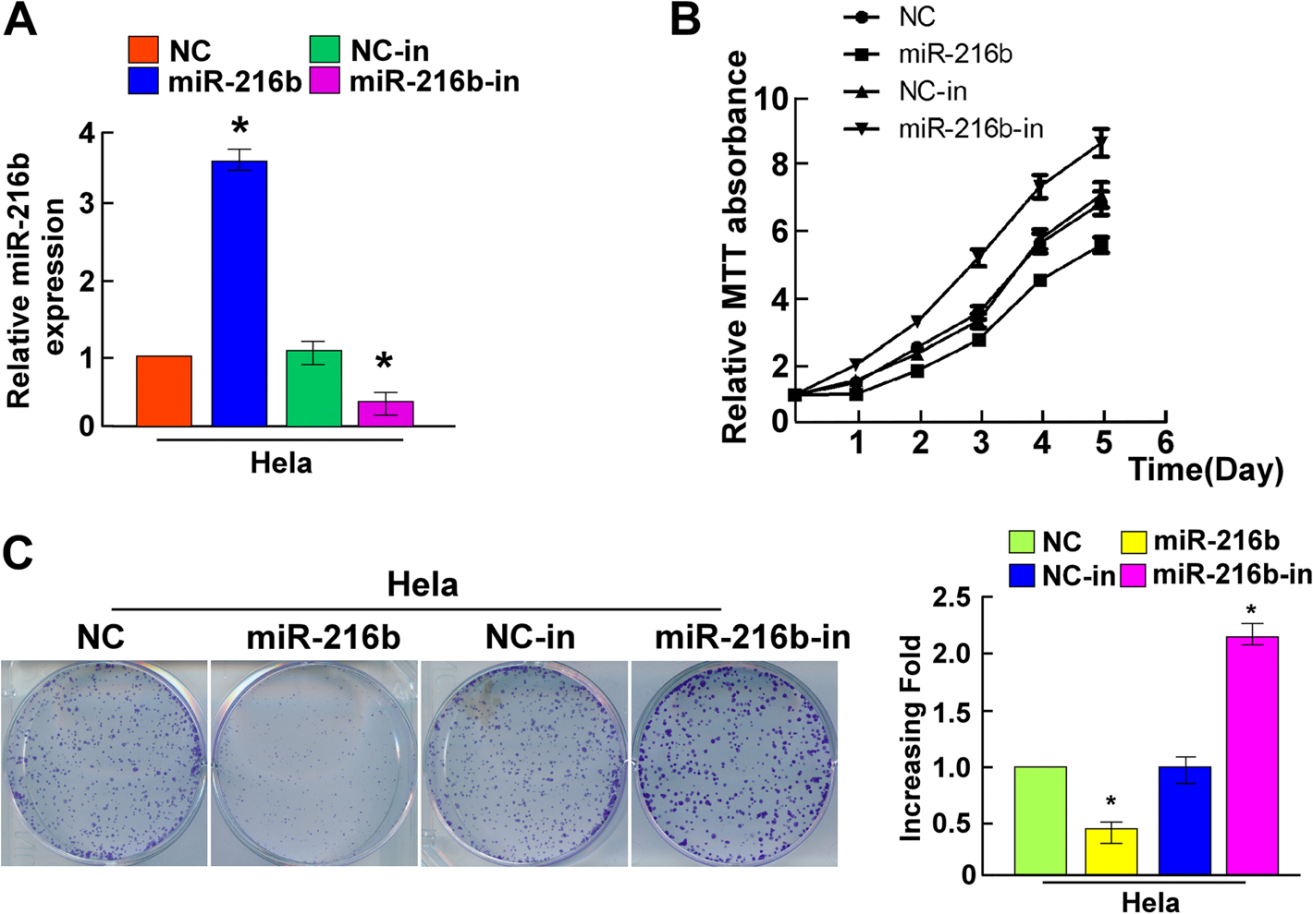
Ectopic miR-216b suppresses the proliferation of HeLa cells. **a** Effect of miR-216b mimics and inhibitors on miR-216b expression. Transfection of miR-216b mimics dramatically elevated miR-216b level and miR-216b inhibitors decreased miR-216b expression in HeLa cells. **b** MTT assay showed that ectopic miR-216b suppressed the proliferation of indicated HeLa cells, whereas miR-216b inhibitors stimulated cell proliferation. **c** Representative micrographs (left) and quantifications (right) of crystal violet stained cell colonies formed by the indicated HeLa cells, 10 days after cell inoculation. Effects of ectopic miR-216b obviously reduced the colony formation ability of indicated HeLa cells, whereas miR-216b inhibition dramatically increased colony formation. Colonies containing >50 cells were counted. * *P* < 0.05

Since there was evidence that miR-216b could suppress tumor growth [21, 22], we further explored the effect of miR-216b on proliferation capacity in HeLa cells. We constructed the miR-216b overexpression and miR-216b inhibited cell model by transfecting miR-216b mimics (miR-216b) and miR-216b inhibitors (miR-216b-in) into HeLa cells, respectively (Fig. 2a), and the cell proliferation ability was analyzed using MTT assay and colony formation assay. We found that ectopic expression of miR-216b could lead to a dramatic decrease in cell proliferation ability. MTT analysis showed that in miR-216b mimics transfected group, cell proliferation ability was suppressed compared to the NC group (Fig. 2b, *P*<0.05 on day 5 and day 6). Colony formation assay showed a significant decrease of cell colonies on day 10, with a dramatic decrease in relative colony number compared with the NC group (*P* < 0.05, Fig. 2c). On the contrary, when miR-216b was inhibited, cell proliferation ability was enhanced (Fig. 2b, *P*<0.05 on day 4, 5 and 6), and there was a dramatic increase of colony number in miR-216b-in group compared with the NC-in group (*P* < 0.05, Fig. 2c). These results confirmed that miR-216b could inhibit proliferation of cervical cancer cells.

### MiR-216b inhibits cell proliferation by regulating CyclinD1, c-myc, p21, p-Rb and LEF1 expression

**Fig. 3:**
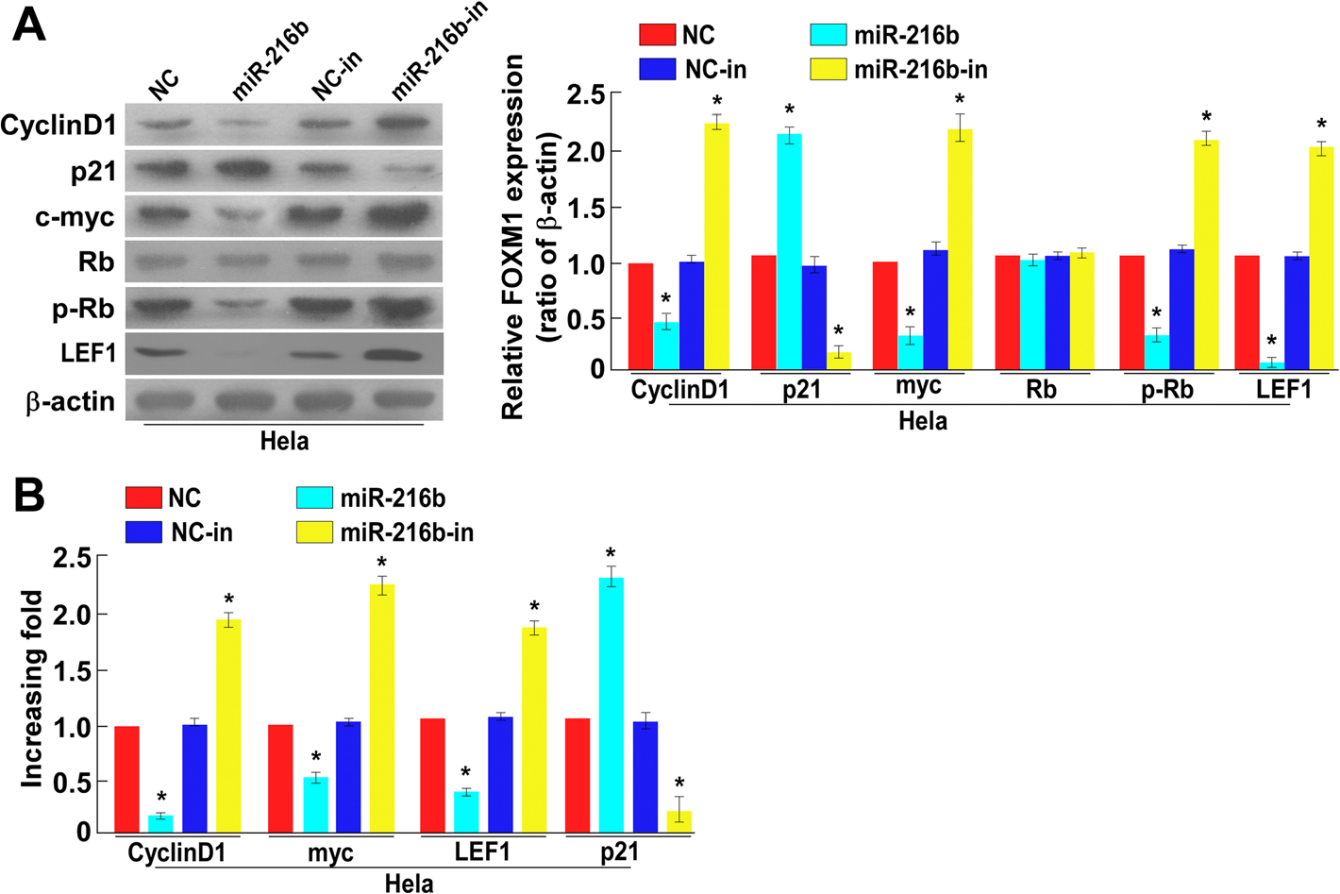
MiR-216b decreases cyclinD1, c-myc, p-Rb and LEF1 level and enhances p21 expression. **a** Western blotting analysis showed that miR-216b mimics decreased the relative expression of cyclin D1, c-myc, LEF1 and phosphorylated Rb (p-Rb) but increased p21 level in HeLa cells 48 h after transfection, and miR-216b inhibitors had the opposite effect, as normalized by β-actin. * *P* < 0.05. **b** Real-time PCR analysis of *cyclin D1*, *p21, myc* and *LEF1* (lymphoid enhancer-binding factor 1) at transcriptional level showed that ectopic miR-216b could significantly decrease *cyclin D1*, *myc* and *LEF1* but increase *p21* mRNA whereas miR-216b inhibitors had the opposite effect. * *P* < 0.05

Because MTT and colony formation assay results suggested that miR-216b may affect the cell cycle of cervical cancer cells, we therefore examined miR-216b effect on cell cycle by flow cytometry, and the results showed that miR-216b could decrease the ratio of cells in S period and miR-216b-in had the opposite effect (Additional file 1: Figure S1). To elucidate the key factors and possible targets of miR-216b in cell proliferation, we further explored the effect of miR-216b on expression of cell cycle related factors, including cyclinD1, p21, c-myc, LEF1, Rb and p-Rb using Western blotting analysis. We found that ectopic expression of miR-216b could dramatically decrease the expression of cyclinD1, c-myc, LEF1 and p-Rb, whereas at the same time, significantly increase the expression level of p21 (*P* < 0.05, Fig. 3a). Accordingly, when HeLa cells were treated with miR-216b inhibitors, a significant decrease in p21 and an increase in cyclinD1, c-myc, LEF1 and p-Rb expression were observed (*P* < 0.05, Fig. 3a and b). These results indicated that miR-216b inhibit cell proliferation by suppressing cyclinD1, c-myc, LEF1 and p-Rb and enhancing p21 expression, and all of which were important regulators or targets of FOXM1 [26–28], suggesting that miR-216b may function by regulating FOXM1. Since FOXM1 has been previously proved to be a downstream component of Wnt/β-catenin signal pathway [29], we further analyzed the mRNA level of Wnt/β-catenin downstream target genes, *cyclinD1*, *p21, myc* and *LEF1* using qRT-PCR. The results showed that ectopic miR-216b could suppress mRNA level of *cyclinD1*, *p21, myc* and *LEF1*, and miR-216b inhibitors had the opposite effect (*P* < 0.05). So miR-216b can inhibit cell proliferation by regulating FOXM1-related cell cycle factors.

### MiR-216b directly targets FOXM1

**Fig. 4:**
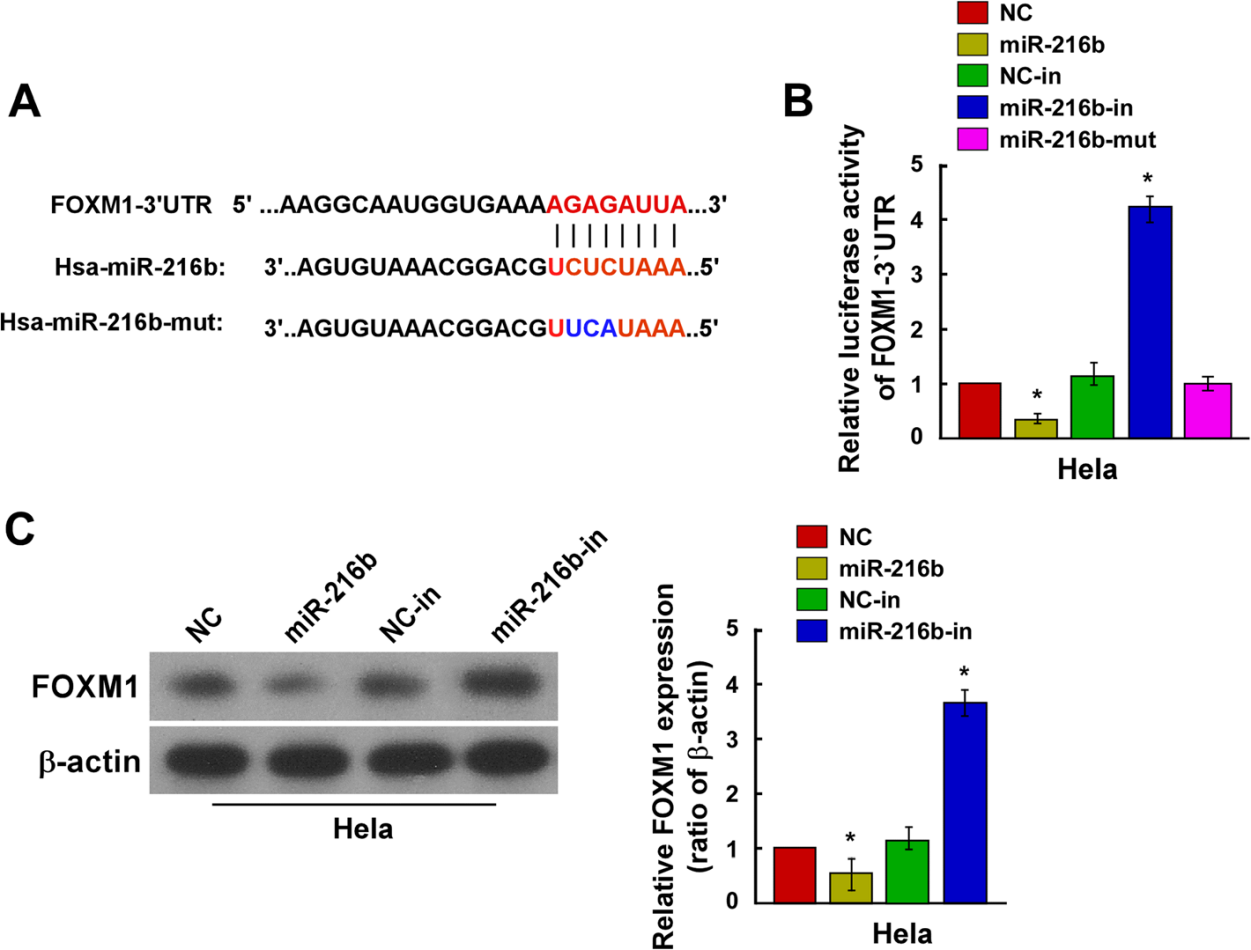
MiR-216b directly targets the 3′-UTR of *FOXM1* mRNA. **a** Schematic representation of the mature miR-216b sequence, putative miR-216b target site in the 3′-UTR of *FOXM1* mRNA, and a mutant of miR-216b containing three altered nucleotides in the FOXM1 binding site (miR-216b-mut). **b** Luciferase assay of PsiCHECK2-FOXM1–3’UTR reporter cotransfected with miR-216b mimics, miR-216b inhibitors or miR-216b mutant in HeLa cells. MiR-216b mimics significantly repressed the luciferase activity of the PsiCHECK2-FOXM1 3′-UTR-luciferase reporter, whereas miR-216b inhibitors showed the opposite effect. MiR-216b mutant had no obvious effect on the luciferase activity of *FOXM1* 3′-UTR reporter. NC was HeLa cells transfected with mimic control. NC-in was HeLa cells transfected with negative control inhibitors. * *P* < 0.05 compared with NC. **c** Western blotting analysis showed the relative expression levels of FOXM1 protein in HeLa cells transfected with miR-216b mimics or miR-216b inhibitors, compared with corresponding control cells (NC and NC-in), 48 h after transfection. MiR-216b mimics suppressed FOXM1 expression and miR-216b inhibitors increased FOXM1 protein level. β-actin served as an internal control. * *P* < 0.05

It has been found that in cervical cancer cells, miR-216b level had an opposite trend of variation against FOXM1 expression. Function analysis showed that multiple FOXM1-related key factors like CyclinD1, c-myc, p-Rb and LEF1 expression can be regulated by miR-216b. We thus suspected that FOXM1 may be a direct target of miR-216b. By using online miRNA target prediction databases including Targetscan and microRNA.org, etc., we found that miR-216b could directly target FOXM1 by binding to its 3′-UTR (position 254–261, AGAGAUUA), as shown in Fig. 4a. Therefore, to examine whether or not miR-216b mediated- FOXM1 down-regulation was effected via the 3′-UTR region, we constructed the psiCHECK2 luciferase reporter vector containing *FOXM1* 3′-UTR, and used a dual luciferase reporter assay system, to clarify the relationship between miR-216b and FOXM1. Western blotting results showed that ectopic expression of miR-216b in HeLa cells significantly repressed the FOXM1 protein level and the luciferase activity of the *FOXM1* 3′-UTR-luciferase reporter, compared to the mimics negative control (NC) (Fig. 4b and c, *P* <0.05), and accordingly, down-regulation of miR-216b (miR-216b-in) led to a significant increase of FOXM1 protein expression and the luciferase activity of the *FOXM1* 3′-UTR-luciferase reporter (Fig. 4b and c, *P* <0.05). By contrast, ectopic expression of miR-216b with a mutant *FOXM1* 3′-UTR binding site (miR-216b-mut) had no obvious effect on the activity of the *FOXM1* 3′-UTR -luciferase reporter (Fig. 4b, *P* <0.05). Therefore, FOXM1 is a direct target of miR-216b.

### MiR-216b inhibits cell proliferation by repressing endogenous FOXM1 in cervical cancer

**Fig. 5:**
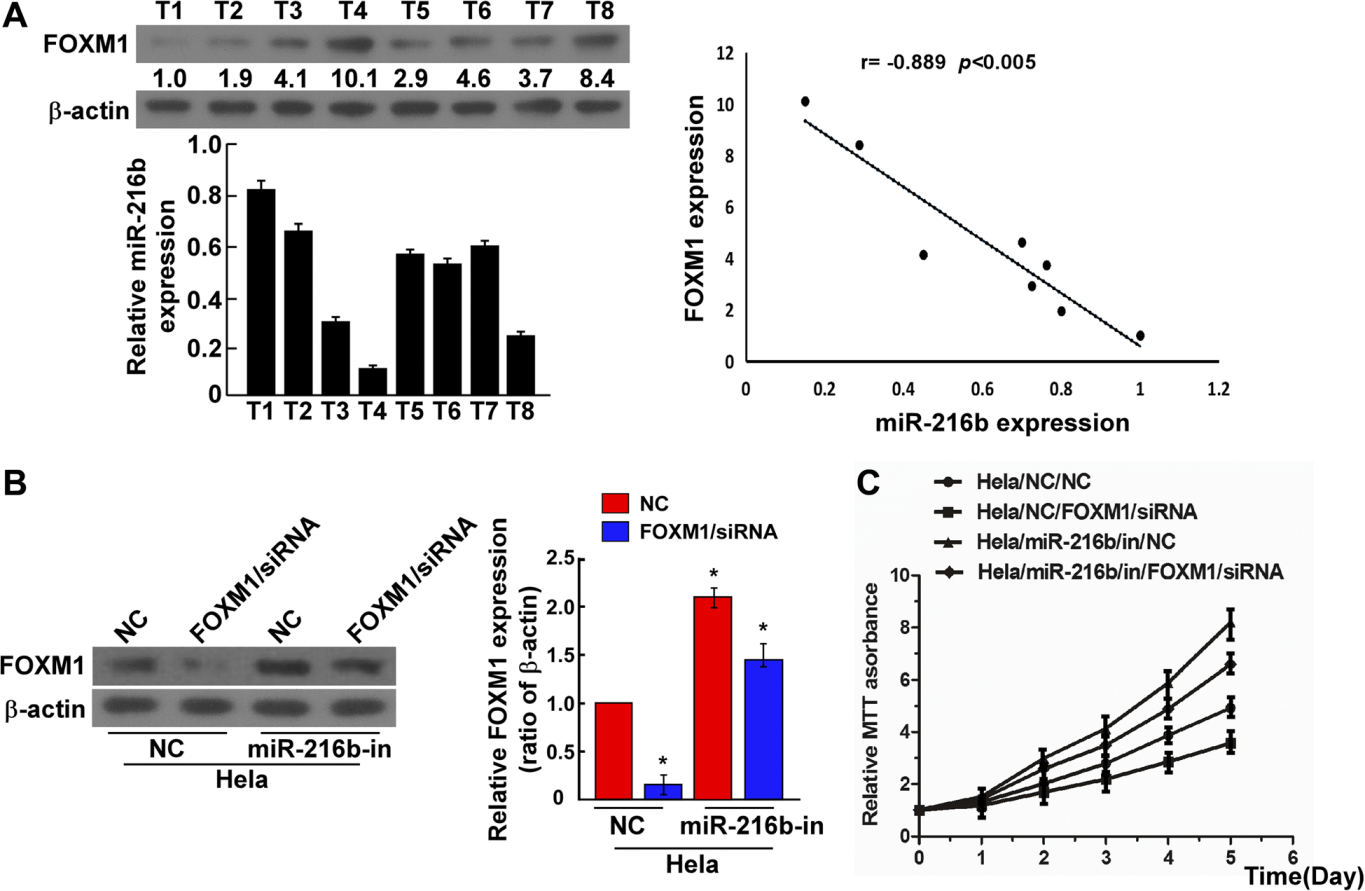
MiR-216b suppresses HeLa cell proliferation by inhibiting FOXM1. **a** The expression levels of FOXM1 (upper left) and miR-216b (bottom left) in cervical cancer tissues of 8 patients were determined by western blotting and real-time RT-PCR, respectively. The relative band intensity values of FOXM1 normalized by β-actin were shown above the β-actin bands. The levels of miR-216b and FOXM1 in cervical cancer tissues were negatively correlated. Correlation analysis (right) confirmed the negative correlation between miR-216b and FOXM1. **b** FOXM1 expression was suppressed by FOXM1-siRNA in both negative control HeLa cells (NC) and miR-216b inhibitors transfected cells (miR-216b-in). Normalized FOXM1 expression was shown. HeLa cells were transfected with or without FOXM1-siRNA and miR-216b inhibitors and measured by western blotting analysis. β-actin served as an internal control. * *P* < 0.05. **c** MTT assay of cell proliferation in FOXM1-silenced HeLa cells. FOXM1-siRNA transfection suppressed the proliferation of HeLa cells, whereas in miR-216b inhibitors transfected cells, the enhanced cell proliferation could be repressed by FOXM1 silencing

To further elucidate miR-216b-FOXM1 regulation relationship, we analyzed FOXM1 and miR-216b level in clinical cervical cancer tissues using western blotting and qRT-PCR analysis (Fig. 5a). The results demonstrated that in all 8 cases, when relative miR-216b level was high, FOXM1 expression was suppressed and low miR-216b showed the opposite effect (Fig. 5a, *P* <0.05). Correlation analysis showed the coefficient *r* between miR-216b and FOXM1 was −0.805 (*P* < 0.01, Fig. 5a), confirmed the negative regulatory relationship. To evaluate the effect of FOXM1 on cell proliferation, we suppressed endogenous FOXM1 expression with its specific siRNA. Western blotting analysis showed that endogenous FOXM1 expression can be successfully inhibited by FOXM1/siRNA in mimics negative control (NC) transfected cells (*P* < 0.05 compared with siRNA negative control, Fig. 5b). But when HeLa cells were transfected previously with miR-216b inhibitor (miR-216b-in), expression of FOXM1 was significantly enhanced in both siRNA negative control and FOXM1/siRNA transfected cell groups, although FOXM1 level could still be suppressed to some extent by its siRNA (Fig. 5b, *P* <0.05). The results indicated that miR-216b could repress endogenous FOXM1 expression. The MTT results indicated that silencing FOXM1 suppressed the proliferation of HeLa cells (Fig. 5c). On the contrary, in miR-216b-in cells, cell proliferation was significantly promoted, whereas in FOXM1 siRNA transfected cells, the effect was less obvious (*P* < 0.05, Fig. 5c). These data suggested that repressing miR-216b could stimulate cervical cancer cell proliferation, whereas silencing endogenous FOXM1 expression could reverse this effect. Therefore, miR-216b inhibits cell proliferation by repressing endogenous FOXM1 expression.

### Higher level of miR-216b is associated with better prognosis in cervical cancer patients

**Fig. 6:**
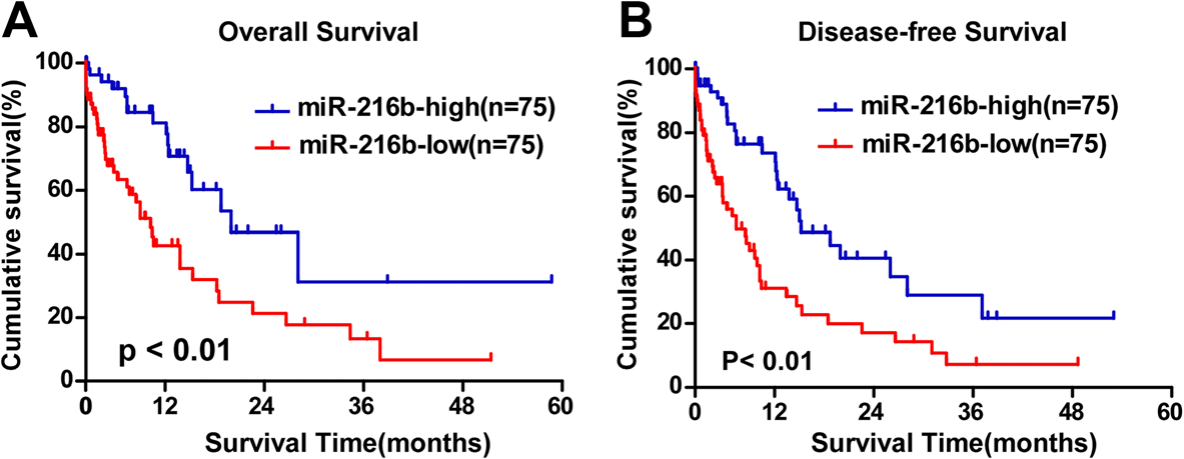
High level of miR-216b expression is related to better prognosis of cervical cancer patients. **a** Overall survival plot of 150 cervical cancer patients stratified by expression of miR-216b. Survival curves of 75 miR-216b-high and 75 miR-216b-low patients were drawn using the Kaplan-Meier method. Higher expression of miR-216b was associated with better overall survival (*P* < 0.01). **b** Disease free survival plot of 150 cervical cancer patients stratified as 75 miR-216b high and 75 miR-216b low patients. Higher expression of miR-216b was more possibly associated with disease free survival compared with miR-216b low patients (*P* < 0.01)

We have proved that FOXM1 level is associated with poor prognosis in early-stage cervical cancer patients in our previous studies [3], so we deduced that miR-216b may also be related to prognosis of cervical cancer patients. Therefore, we choose 150 samples (75 miR-216b levels high and 75 miR-216b low) of cervical cancer patients for survival analysis. The real-time PCR detection results were shown in Additional file 2: Figure S2. No statistical age difference existed between miR-216 high and miR-216b low groups (Table 2). The results confirmed that high expression of miR-216b was related to earlier FIGO stage (I, II) and better survival status in cervical cancer patients (*P* < 0.01, Fig. 6, Table 2). More miR-216 high samples were histological SCC, indicating that SCC may tend to express higher level of miR-216b (*P* < 0.05, Table 2). Notably, the overall survival and disease free survival in miR-216b high group were also significantly better than miR-216b low group (*P* < 0.01, Fig. 6). Therefore, we found that higher level of miR-216b is associated with better prognosis in cervical cancer patients, and miR-216b has the potential of being a prognostic biomarker.

**Table 2:** Correlation between the clinicopathological features and expression of miR-216

Patient characteristics		miR-216 expression		*P*-value
		Low	High	
Age (years)	≤55	36	40	0.627
	>55	39	35	
FIGO stage	I, II	10	41	< 0.001
	III, IV	65	34	
Histology	Squamous	54	67	= 0.012
	Others	21	8	
Survival status	Alive	9	46	< 0.001
	Dead	66	29	

Patient number of miR-216b-low and miR-216b-high groups and the corresponding proportion (%) were shown. The clinicopathological differences between miR-216b-low and miR-216b- high groups were analyzed using Pearson’s χ2 test

## Discussion

FOXM1 is involved in multiple biological process including cell proliferation, differentiation, growth, migration and invasion [26, 30, 31]. Its overexpression is implicated to play an important role in pathogenesis, progression and metastasis of many types of cancers [29–34], including cervical cancer as we have confirmed in our previous study [3]. Although there is report that miR-342-3p could suppress the proliferation and metastasis in cervical cancer by targeting FOXM1 [23], the FOXM1- microRNAs modulation pathways in cervical cancer cells remains to be discovered. Based on our previous study outcomes, to further elucidate the regulation pathway of FOXM1 in cervical cancer, in this study, we screened possible FOXM1 upstream modulator microRNAs using online miRNA target prediction databases including Targetscan and microRNA.org and presumed that miR-216b may regulate FOXM1 expression.

MiR-216b gene has been reported to be involved in several cancers including nasopharyngeal carcinoma, medulloepitheliomas, breast cancer, etc. [21, 22, 35–37]. Although recently it has been reported that miR-216b could inhibit cell growth by targeting FOXM1 in hepatocellular carcinoma and melanoma [38, 39], the role of miR-216b and the relationship between miR-216b and FOXM1 in cervical cancer remains unclear. In the present study, we first screened the expression level of miR-216b in cervical cancer cell lines and clinical samples, and found that miR-216b was down-regulated, suggesting that miR-216b is involved in cervical cancer and may be a tumor suppressor miRNA. We also found that miR-216b level in these cervical cell lines showed a reverse trend of FOXM1 expression (Fig. 1a), indicating that miR-216b may regulate FOXM1 expression. Because the involvement of FOXM1 in tumorigenesis is mainly related to its role in cell-cycle progression and proliferation, and migration and invasion of cervical cancer cells [3], we further explored the cervical cancer cell cycle, cell proliferation and invasion ability after miR-216b overexpression and inhibition. The results showed that overexpression of miR-216b could significantly inhibit cell proliferation in HeLa cells (Fig. 2) and decrease the ratio of cells in S period (Additional file 1: Figure S1), but its effect on tumor invasion and migration was not obvious (data not shown), indicating that miR-216b may regulate FOXM1-related cell proliferation factors, but not FOXM1-related metastasis factors, and there may be other microRNA-FOXM1 pathways involved in FOXM1 related cell metastasis. Further studies will be needed to explore more micorRNA-FOXM1 links that are involved in cervical cancer carcinogenesis.

To elucidate miR-216b targets and to further verify the relationships between miR-216b and FOXM1, we explored the effect of miR-216b on expression of FOXM1 related cell division genes, including the CDK (cyclin-dependent kinases) inhibitor p21 which can be negatively regulated by FOXM1, Rb and p-Rb that could indirectly regulate FOXM1 activity as an upstream regulator of FOXM1, and the CDK regulator cyclin D1 which can be positively regulated by FOXM1, and also the mRNA level of Wnt/β-catenin downstream target genes, *cyclinD1*, *myc* and *LEF1*, as FOXM1 has been previously proved to be a downstream component of Wnt/β-catenin signal pathway [29]. The results proved our deduction. We observed that p21 was up-regulated and Wnt/β-catenin downstream targets including cyclinD1, myc and LEF1 were down-regulated in miR-216b overexpression cells, and up-regulated in miR-216b inhibited cells. Coincident with altered expression of cell-cycle regulators, the phosphorylation level of Rb, a downstream target protein level of CDK and an upstream regulator of FOXM1, was significantly decreased in miR-216b transfected cells and obviously increased in miR-216b inhibited cells (Fig. 3a), further confirming that miR-216b can affect the proliferation of cervical cancer cells by regulating FOXM1 related cell division factors. Using dual luciferase reporter system, we proved that miR-216b directly bind to FOXM1 3′- UTR. Therefore, FOXM1 is a direct target of.miR-216b. Further analysis confirmed that miR-216b inhibits cell proliferation by repressing endogenous FOXM1 in cervical cancer cells and tissues, and negative correlation existed between miR-216b and FOXM1 with coefficient *r* − 0.805 (Fig. 5a). These findings indicate that dysregulation of FOXM1 by miR-216b may be an important mechanism underlying cervical cancer tumorigenesis, and future studies should address the detailed molecular mechanisms behind the role of miR-216b-FOXM1 link in the tumorigenesis of cervical cancer. However, the interaction of microRNAs and transcription factors in tumors contains very complicated networks, and the relationship of miR-216b-FOXM1 is only been reported recently [38, 39], and not been included in the recent published work of systematic -omic evaluation of cervical cancer by the Cancer Genome Atlas Project (TCGA) [40]. The reason may lies in different control and hierarchical clustering. In our study, the relative T/ANT (cancer tissues/adjacent non-cancer tissues) ratio of FOXM1 [3] and miR-216b expression was examined in cervical cancer tissues, whereas in TCGA studies, cancer tissues/normal controls were compared. The change of p21, myc expression and Wnt/β-catenin signal pathway in cervical cancer were also revealed in TCGA study, using squamous/adenocarcinomas and HPV positive/negative hierarchy, whereas in our study, most of the cancer tissues were squamous and HPV positive. We believe our study will further enrich and helps to understand the molecular modulation mechanism of tumor associated genes and factors in cervical cancer.

FOXM1 has been proved to be a prognostic factor for poor survival in patients with early-stage cervical cancer [3]. Since miR-216b targets and suppresses FOXM1, it may also be related to the prognosis of cervical cancer patients. We then evaluated the role of miR-216b in the prognosis of 150 patients (121 SCC and 29 other types) using Kaplan-Meier survival analysis. It was confirmed that as a contrast to FOXM1, high expression of miR-216b was related to earlier FIGO stage, better histological type and better survival status in cervical cancer patients (Table 2, Fig. 6). However, the overall mortality rate was relatively higher than that in FOXM1 study [3], because more late-stage patients who receive only chemo- radiotherapy or no treatment were enrolled in this study (Table 1). One reason that more late-stage patients were collected was that their lesions were clearer and tumor specimen was easier to obtain for miR-216b detection. And with more late-stage cancer tissues, the trend that miR-216b expression decreased with the FIGO stage was more obvious, consistent with reports of other tumors [21, 35–37]. Moreover, more late-stage specimens can better display the value of miR-216b in cervical cancer development and prognosis as a biomarker. Despite of the high mortality, we still found that higher level of miR-216b was associated with both better overall survival and better disease-free survival than miR-216b low level patients (*P* < 0.01, Fig. 6). As far as we know, this is the first time that miR-216b is reported to be correlated with better prognosis of cervical cancer patients. High level of miR-216b in cervical cancer patients indicated not only longer survival time but also longer disease-free time. Therefore, miR-216b may also be a potential prognostic marker for cervical cancer.

## Conclusions

In summary, the current study shows that miR-216b is down-regulated in cervical cancer cells and tissues, and could inhibit the proliferation ability of cervical cancer cells. It is also shown that FOXM1 expression is suppressed by miR-216b via direct binding to FOXM1 3′-UTR. Besides, miR-216b could inhibit cell proliferation by regulating FOXM1 related Wnt/β-catenin signal pathway. High level of miR-216b is related to better prognosis in cervical cancer patients and may serve as a potential prognostic marker. The newly found miR-216b/FOXM1 link provides a clue to the discovery of the potential mechanism for FOXM1 dysregulation and cervical cancer tumorigenesis. Modulation of FOXM1 expression through miR-216b regulation shed new lights on the molecular intervention therapy for cervical cancer.

## Additional files

Additional file 1: Figure S1. MiR-216b suppresses cell proliferation by suppressing cell cycle. (TIFF 69 kb)

Additional file 2: Figure S2. Quantitative RT-PCR detection of 150 cervical cancer samples divided them into miR-216 high and miR-216 low groups. (TIFF 32 kb)

## Abbreviations

CDK: Cyclin-dependent kinases
FIGO: International Federation of Gynecology and Obstetrics
FOXM1: forkhead box M1
LEF: Lymphoid enhancer-binding factor
miR-216b: microRNA 216b
miRNA: microRNA
MTT: Methyl thiazolyl tetrazolium
PBS: Phosphate buffered saline
PVDF: Polyvinylidene fluoride
qPCR: quantitative polymerase chain reaction
Rb: retinoblastoma
RT-PCR: Reverse transcription polymerase chain reaction
SCC: Squamous cell carcinoma
siRNA: small interfering RNA
UTR: Untranslating region
