# Supplementary figures and images for: MiR-216b inhibits cell proliferation by targeting FOXM1 in cervical cancer cells and is associated with better prognosis
Source: BMC Cancer. 2017 Oct 4;17:673. doi: 10.1186/s12885-017-3650-5 (PMC5628450; doi:10.1186/s12885-017-3650-5)

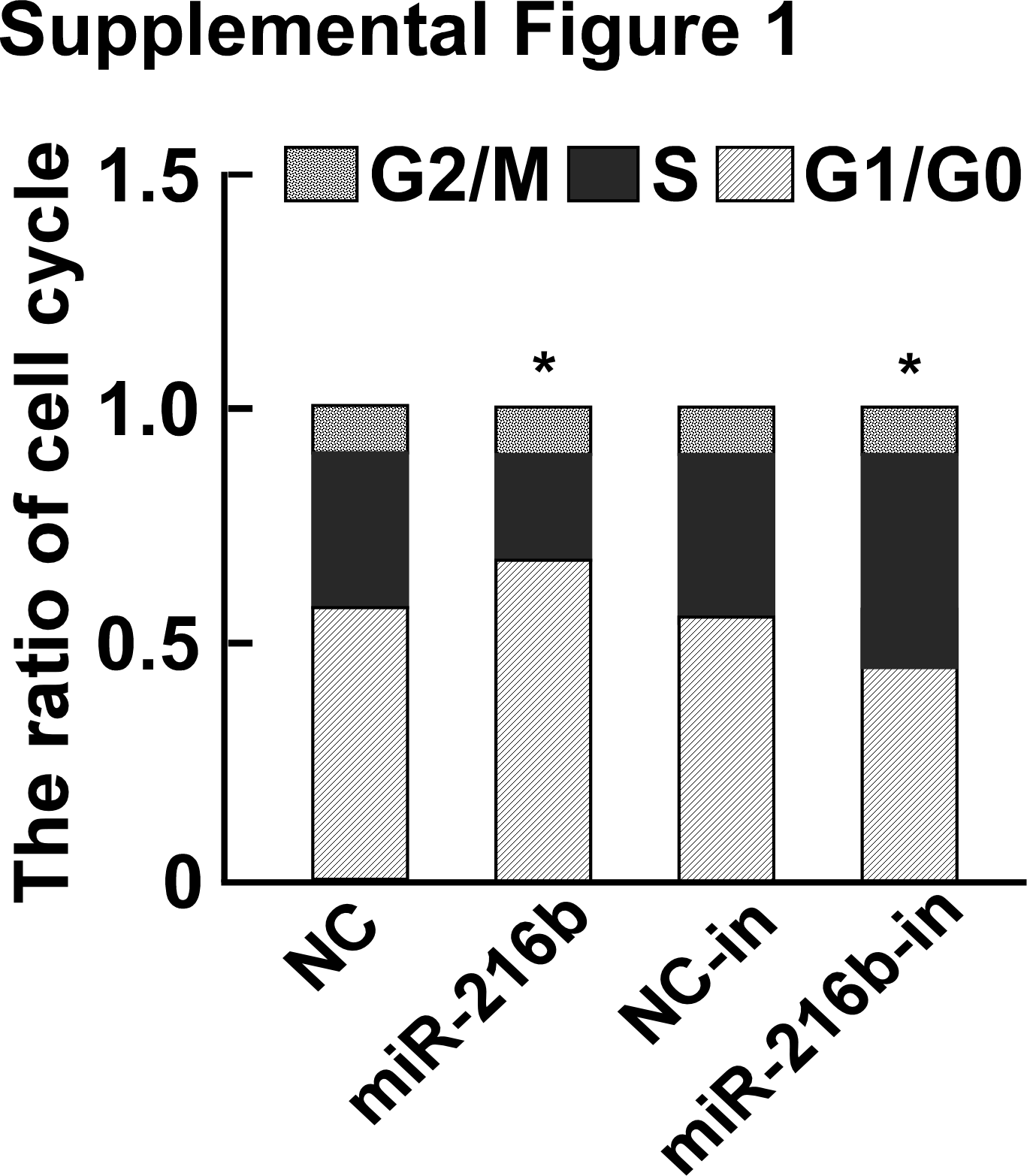

Supplement: Supplementary file 1 — MiR-216b suppresses cell proliferation by suppressing cell cycle. (TIFF 69 kb) [file 12885_2017_3650_MOESM1_ESM.tif]
